# Multiple Cystic Components With Bleeding Are Important Diagnostic Findings for Encapsulated Papillary Carcinomas of the Breast: A Case Report

**DOI:** 10.7759/cureus.104864

**Published:** 2026-03-08

**Authors:** Takuro Uchida, Shoji Oura

**Affiliations:** 1 Department of Surgery, Kishiwada Tokushukai Hospital, Kishiwada, JPN

**Keywords:** breast cancer, encapsulated papillary carcinoma, intracystic bleeding, invasive micropapillary carcinoma, multiple cystic components

## Abstract

Encapsulated papillary carcinoma (EPC) is a rare papillary breast lesion with known favorable biology, but is not fully understood of its image findings. A 78-year-old woman with a screening mammography-detected breast mass was referred to our hospital. Mammography showed an oval mass, 4.3 cm in size, with circumscribed margins in her left breast. Ultrasound showed that the mass consisted of high-echo areas and many cystic areas. Magnetic resonance imaging (MRI) of the mass showed weak high signals in some areas on T1-weighted images and mixed high and low signals with fluid-fluid level formation on T2-weighted images. Core needle biopsy pathologically showed cancer cells growing in low papillary and papillary fashions and had microvoids around cancer cells, leading to the suspected diagnosis of invasive micropapillary carcinoma (IMPC). The patient, therefore, underwent a partial mastectomy and a sentinel node biopsy. The tumor was completely encapsulated with a fibrous capsule and consisted of cancer cells growing in cribriform, papillary, and low papillary fashions with cystic structures and intracystic bleeding. Immunostaining showed estrogen receptor positivity (Allred score 8), progesterone receptor positivity (Allred score 7), human epidermal growth factor receptor type 2 equivocality (fluorescence in-situ hybridization negative), a Ki-67 labelling index of 17%, and no myoepithelial cells, leading to the final diagnosis of EPC. The patient recovered uneventfully and has been well on adjuvant aromatase inhibitor therapy for 14 months. Diagnostic physicians should note that multiple cystic components with bleeding in the mass may be an important diagnostic clue to differentiate EPC from other papillary or pseudopapillary breast disorders, including IMPC.

## Introduction

Various benign and malignant papillary lesions occur in the breast [1]. Encapsulated papillary carcinoma (EPC) is a rare papillary breast lesion and is pathologically judged as invasive carcinoma due to the lack of myoepithelial cells, which non-invasive ductal carcinoma of the breast has. EPCs, however, are enveloped by a fibrous capsule and generally show excellent postoperative clinical outcomes. The World Health Organization (WHO), therefore, classifies EPC in a category equivalent to non-invasive carcinoma in order to prevent over-treatment when lacking frank invasion [2].

EPC is most often detected as a mass, but is also sometimes detected by bloody nipple discharge. In addition, EPCs with bloody nipple discharge tend to be detected as larger tumors due to their cystic growth patterns.

Breast cancer with papillary structures generally shows favorable clinical outcomes due to its high differentiation degree. Invasive micropapillary carcinoma (IMPC) also has the term papillary in its name, but has pseudopapillary structures pathologically. IMPC is not a true papillary lesion and often shows poor clinical outcomes due to both the high nuclear grades and node positivity, despite the high estrogen receptor positivity [3,4]. It, therefore, is clinically very important for diagnostic physicians to properly differentiate IMPC from other breast disorders with papillary structures.

Papillary lesions, not limited to the breast disorders, have some kind of microvoids, small areas free of cellular components, in the papillary structures, which make ultrasound backscattering and generate hyperechoic patterns [5,6]. In short, all disorders with papillary or pseudopapillary structures generally have hyperechoic patterns. Therefore, unknown typical images specific to EPC make it difficult for us to distinguish it from other breast papillary lesions, only with echo findings.

We experienced this rare EPC with the initial pathological misdiagnosis of IMPC, found multiple cystic components with intralesional bleeding to be a potential clue to EPC, and herein report the correlation between these imaging and pathological findings.

## Case presentation

**Figure 1:**
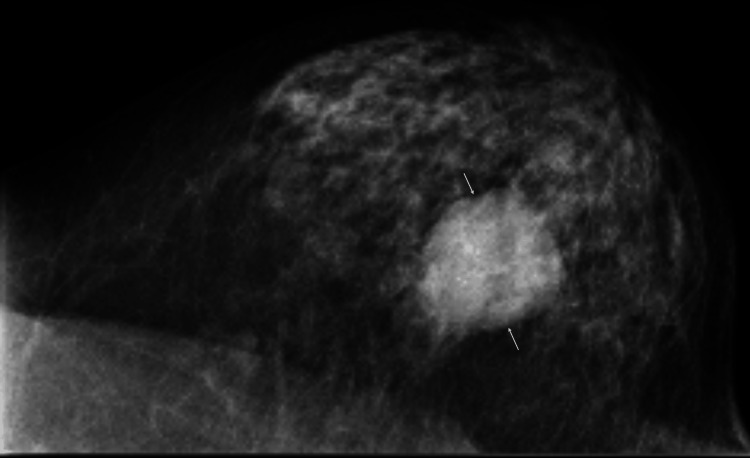
Mammography findings Mammography showed an oval mass with distinct margins (arrows) in the left breast.

A 78-year-old woman without any awareness of breast-related symptoms was found to have a breast mass on annual screening mammography and was referred to our hospital in November 2024, three weeks after the examination, for detailed examination. Mammography showed an oval mass, 4.3 cm in size, with circumscribed margins in her left breast (Figure 1).

**Figure 2:**
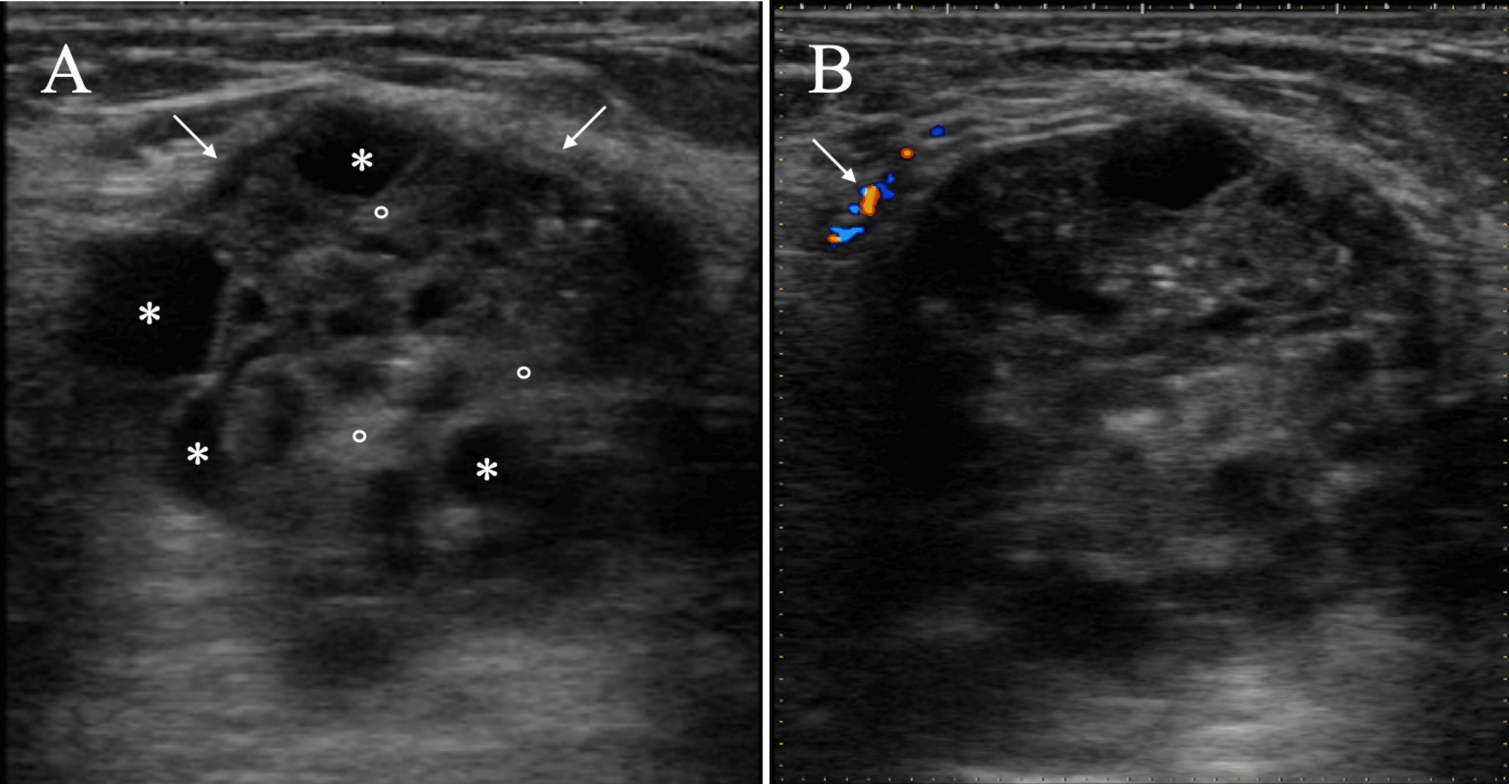
Ultrasound findings Ultrasound showed an oval mass which had distinct margins (A, arrows), a hyperechoic pattern (A, open circles), multiple small cystic areas (A, asterisks), and peritumoral blood flow (B, arrow).

Ultrasound using a 12 MHz probe also showed a circumscribed oval mass, which consisted of high echo areas and multiple cystic areas, with posterior echo enhancement and peritumoral blood flow (Figure 2). These findings led to the evaluation of Breast Imaging Reporting and Data System Category 4, which suggested cystic component-containing breast disorders such as phyllodes tumor and invasive ductal carcinoma with cystic components.

**Figure 3:**
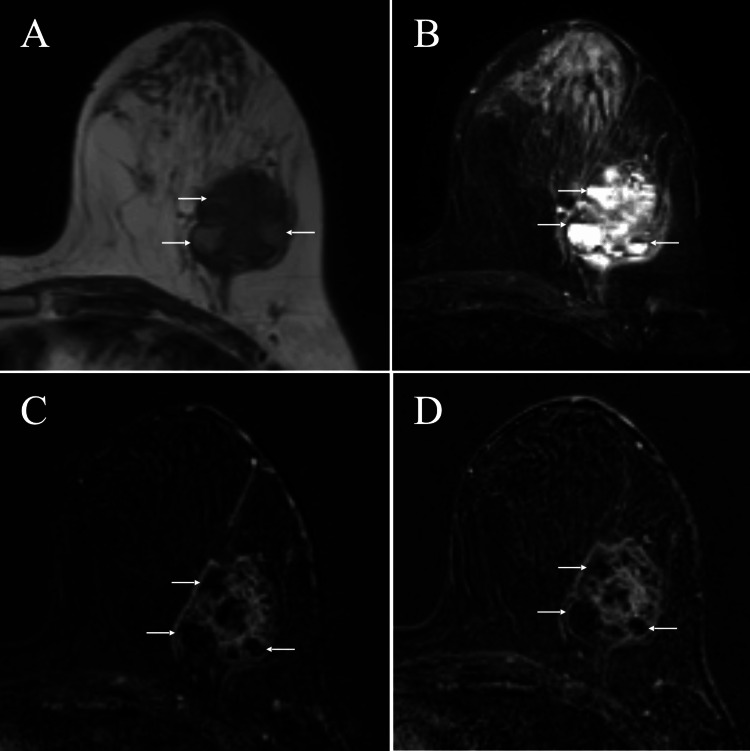
Magnetic resonance imaging (MRI) findings A. MRI of the tumor showed low signals with focal weak high signals (arrows) on T1-weighted images. B. MRI of the tumor showed high and low signals with fluid-fluid level formation (arrows) on T2-weighted images. C. Initial phase dynamic studies, i.e., two minutes, showed a fast enhancement in the solid areas and no enhancement (arrows) in the weak high signal areas on T1-weighted images. D. Delayed phase dynamic studies, i.e., six minutes, showed a plateau enhancement in the solid areas and retained no enhancement (arrows) similar to those of initial phase studies.

Magnetic resonance imaging (MRI) of the mass showed weak high signals in some areas on T1-weighted images, mixed high and low signals with fluid-fluid level formation on T2-weighted images, and a fast/plateau pattern on dynamic studies using gadolinium (Figure 3).

**Figure 4:**
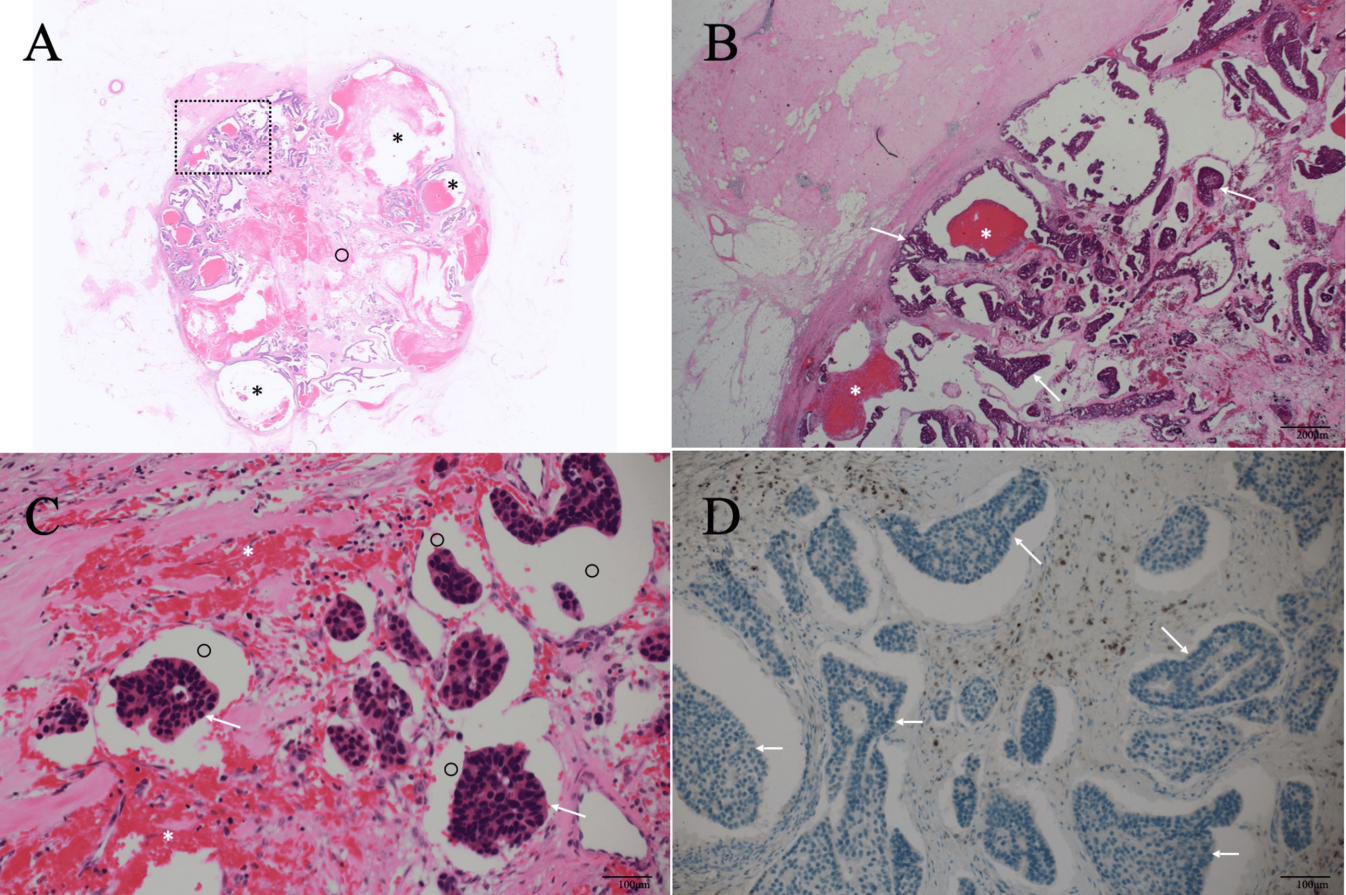
Pathological findings A. Low magnified view showed an oval tumor with many cystic areas (asterisks) and fibrous component-rich areas (open circle). B. Magnified view of the dotted square area in Figure A showed cancer cell clusters (arrows) and bleeding (asterisks) in the cystic areas. C. Magnified view showed cancer cell clusters (arrows) with micro voids (open circles). D. Immunostaining showed no p63 positive cells in cancer cell clusters (arrows).

These imaging findings made us highly suspect the mass to be breast cancer and to do a core needle biopsy on the mass. Pathological study of the four core needle biopsy specimens using a 14-gauge needle showed atypical cells growing in low papillary and papillary fashions with psammoma body deposition and microvoids around cancer cells, leading to the suspected diagnosis of IMPC. Immunostaining showed estrogen receptor (ER) positivity (Allred score 8), progesterone receptor (PgR) positivity (Allred score 7), human epidermal growth factor receptor type 2 equivocality, and a Ki-67 labelling index of 14%. The patient, therefore, underwent a partial mastectomy and a sentinel node biopsy, revealing node negativity with negative surgical margins on frozen section. Postoperative pathological study showed a distinct mass, 30 mm in size, which was completely encapsulated with a fibrous capsule and consisted of cancer cells growing in cribriform, papillary, and low papillary fashions, i.e., nuclear grade 1, with cystic structures and intracystic bleeding. Immunostaining showed the same findings about ER, PgR, and HER2 (fluorescence in-situ hybridization negative) as those of the core needle biopsy specimens and clarified a Ki-67 labelling index of 17% and no myoepithelial cells in cancer cell clusters by p63 staining (Figure 4).

These pathological findings led to the final diagnosis of EPC. The patient recovered uneventfully and was discharged on the second day after the operation. Because it is unclear whether adjuvant radiotherapy (RT) and endocrine therapy can be omitted in margin-negative and ER-positive EPC treated with breast-conserving surgery, the patient received RT and has been well on aromatase inhibitor therapy for 14 months.

## Discussion

Papillary lesions of the breast include intraductal papilloma, papillary ductal carcinoma in situ, encapsulated papillary carcinoma, solid papillary carcinoma (in situ and invasive), and invasive papillary carcinoma [1]. All these papillary lesions have basically intraductal components, which can cause intraductal bleeding sometimes followed by bloody nipple discharge. As mentioned above, these lesions generally have hyperechoic patterns [5,6]. Diagnostic physicians, therefore, can hardly differentiate these papillary disorders only with ultrasound and clinical findings.

IMPC consists of predominant, i.e., >90%, hollow or morula-like aggregates in empty stromal spaces [7]. In addition, cancer cell clusters have an inside-out growth pattern, which means cancer cell lumens facing stromal side [7]. IMPC has the word "papillary" in its disease name, but does not have true ductal structures connected to the nipple. These characteristics imply IMPC is not a true papillary lesion, but a pseudopapillary lesion [7]. IMPC, therefore, hardly presents bloody nipple discharge, unlike other papillary disorders of the breast.

The tumor had focal weak high signal areas on T1-weighted images. In addition, the tumor had multiple fluid-fluid levels between the high and low signals in its cystic parts on T2-weighted images. It is well known that blood shows low signals on T2-weighted images when having oxyhemoglobin, deoxyhemoglobin, and intracellular methemoglobin, i.e., fresh blood, and presents high signals when having extracellular methemoglobin, i.e., old blood [8]. Fluid-fluid level formation, therefore, can be consistent with hemorrhagic components. Dynamic studies further support this evaluation due to the absence of enhancement areas both in these high and low signal areas.

Papillary lesions often present as masses with a mixture of cystic and solid areas on ultrasound. The vast majority of mixed cystic and solid tumors have predominant cystic areas and focal tumor areas. This type of papillary lesions generally have distinct cystic margins except for solid areas with extra-capsular invasion. In addition, these papillary lesions often have masses with smooth edges in malignant cases and those with sharp edges in benign cases. On the other hand, some papillary lesions have predominantly solid areas with focal cystic areas, often due to the aggressive tumor characteristics, e.g, central necrosis. This type of papillary lesions, however, generally have one or two cystic areas and extremely rarely have several or multiple cystic areas [9,10].

We operated on the patient under the preoperative pathological diagnosis of IMPC. If the diagnosis of EPC had been made preoperatively, we might have considered omitting the sentinel lymph node biopsy. This EPC, however, had multiple cystic components with bleeding in the tumor, which markedly differ from the image findings of IMPC [11-13]. Breast specialists, therefore, should take these image findings about EPC into consideration in the differential diagnosis of papillary or pseudopapillary lesions of the breast.

## Conclusions

Multiple cystic components in the mass may be a helpful clue to differentiate EPC from other papillary or pseudopapillary breast disorders, including IMPC. Bleeding within the cystic components may also be useful in differentiating IMPC from other papillary lesions. Diagnostic physicians should note that pathological evaluation is most important in diagnosing EPC, and that other papillary lesions with cystic components may overlap with EPC. In any case, diagnostic physicians should note these image findings about EPC when diagnosing presumed papillary or pseudo papillary lesions of the breast.
